# Progressive Vascular Functional and Structural Damage in a Bronchopulmonary Dysplasia Model in Preterm Rabbits Exposed to Hyperoxia

**DOI:** 10.3390/ijms17101776

**Published:** 2016-10-24

**Authors:** Julio Jiménez, Jute Richter, Taro Nagatomo, Thomas Salaets, Rozenn Quarck, Allard Wagennar, Hongmei Wang, Jeroen Vanoirbeek, Jan Deprest, Jaan Toelen

**Affiliations:** 1 Department of Development and Regeneration, Group Biomedical Sciences, KU Leuven, 3000 Leuven, Belgium; juliojimenez@gmail.com (J.J.); jute.richter@uzleuven.be (J.R.); thomas.1.salaets@uzleuven.be (T.S.); 2 Department of Obstetrics and Gynaecology, Clínica Alemana Universidad del Desarrollo, 7650568 Santiago, Chile; 3 Department of Pediatrics, Ehime Prefectural Central Hospital, 790-0024 Ehime, Japan; ctnagatomo@gmail.com; 4 Respiratory Division, Department of Clinical and Experimental Medicine, University of Leuven, 3000 Leuven, Belgium; Rozenn.Quarck@kuleuven.be (R.Q.); allard.wagenaar@kuleuven.be (A.W.); 5 Department of Obstetrics and Gynecology, Shandong Provincial Hospital Affiliated to Shandong University, 250021 Jinan, China; whmeisd@163.com; 6 Centre for Environment and Health, Department of Public Health and Primary Care, KU Leuven, 3000 Leuven, Belgium; Jeroen.Vanoirbeek@kuleuven.be; 7 Department of Obstetrics and Gynaecology, University College London, WC1E 6HX London, UK; 8 Division of Woman and Child, University Hospitals Leuven, 3000 Leuven, Belgium; jaan.toelen@uzleuven.be

**Keywords:** bronchopulmonary dysplasia, animal models, lung chronic disease, rabbit, lung function

## Abstract

Bronchopulmonary dysplasia (BPD) is caused by preterm neonatal lung injury and results in oxygen dependency and pulmonary hypertension. Current clinical management fails to reduce the incidence of BPD, which calls for novel therapies. Fetal rabbits have a lung development that mimics humans and can be used as a translational model to test novel treatment options. In preterm rabbits, exposure to hyperoxia leads to parenchymal changes, yet vascular damage has not been studied in this model. In this study we document the early functional and structural changes of the lung vasculature in preterm rabbits that are induced by hyperoxia after birth. Pulmonary artery Doppler measurements, micro-CT barium angiograms and media thickness of peripheral pulmonary arteries were affected after seven days of hyperoxia when compared to controls. The parenchyma was also affected both at the functional and structural level. Lung function testing showed higher tissue resistance and elastance, with a decreased lung compliance and lung capacity. Histologically hyperoxia leads to fewer and larger alveoli with thicker walls, less developed distal airways and more inflammation than normoxia. In conclusion, we show that the rabbit model develops pulmonary hypertension and developmental lung arrest after preterm lung injury, which parallel the early changes in human BPD. Thus it enables the testing of pharmaceutical agents that target the cardiovascular compartment of the lung for further translation towards the clinic.

## 1. Introduction

Bronchopulmonary dysplasia (BPD) is a chronic lung disease that represents one of the most important diseases of preterm birth survivors [1]. It affects between 22%–68% of preterm infants and is inversely related to the gestational age at birth [2]. BPD is characterized by an arrested lung development (i.e., decreased alveolar septation), vascular changes (i.e., dysregulated development of pulmonary microvasculature and a variable pulmonary hypertension) and inflammation [3,4]. Significant improvements in perinatal care—including antenatal corticosteroid administration, preterm birth prevention, surfactant therapy and improved ventilation strategies—have increased survival rate, yet have failed to decrease the incidence of chronic lung disease [5]. Infants with BPD are at risk for recurrent and prolonged hospitalizations, higher complications of other pathologies related with prematurity, and lifelong alterations in lung function [1].

Preterm birth and its ensuing injury in the developing lungs impair angiogenesis and alveolarization. This results in pulmonary vascular damage [6,7] and a pathophysiological response that leads to pulmonary hypertension (PH) in up to 25% of the BPD affected infants [8]. Morbidity and mortality are increased in infants who have both BPD and PH, with up to 50% mortality within two years from diagnosis [9].

Our current understanding of the pathophysiology, etiology and treatment of BPD comes from infant studies or animal models [10,11,12,13]. The choice of an animal model in which to investigate novel preventive and therapeutic strategies is difficult, as multiple species and *noxae* are available to induce BPD-like features in the lung tissue. Ideally, a model should have both functional and structural changes in the alveolar, interstitial and vascular compartments that parallel the pathophysiology of human BPD. Mouse models are frequently used because of their low cost, short pregnancy duration, large litters and the availability of transgenic animals. Yet, their perinatal lung development is different to that of humans, as alveolarization takes place after birth [10]. Furthermore the clinical translation of therapeutic interventions in the mouse model proved to be disappointing [14]. Larger animal models, like sheep or non-human primates, are better mimics of fetal and postnatal lung development, but their use entails higher costs and housing demands, and their gestation lasts longer, with only one or two fetuses per pregnancy. In all these models, vascular damage has been characterized [10,12,13,14]. In contrast, rabbits combine elements of the rodent and larger animal models, as they have a relatively short pregnancy duration and a large litter size [11] but also display comparable perinatal lung development to humans. Moreover, their lungs show comparable histological changes and inflammatory responses when exposed to hyperoxia, including alveolar simplification and lung function impairment [15]. To date there is no information about lung vascular damage in this model.

In the present study, we investigate the presence and progression of vascular and parenchymal changes in the lungs of preterm rabbits during sustained hyperoxia. We hypothesize that hyperoxia will cause progressive changes to the vascular compartment of the developing lung that will result in a phenotype of PH. We will use both functional and structural readouts to provide comprehensive data on these changes. This will strengthen the value of the rabbit model both in the study of the physiology and in the testing of experimental therapies before translation towards larger animal models.

## 2. Results

Detailed results are presented in Table 1.

### 2.1. Vascular Changes: Hyperoxia Induces Lung Vasculature Changes and Results in Pulmonary Hypertension

**Figure 1:**
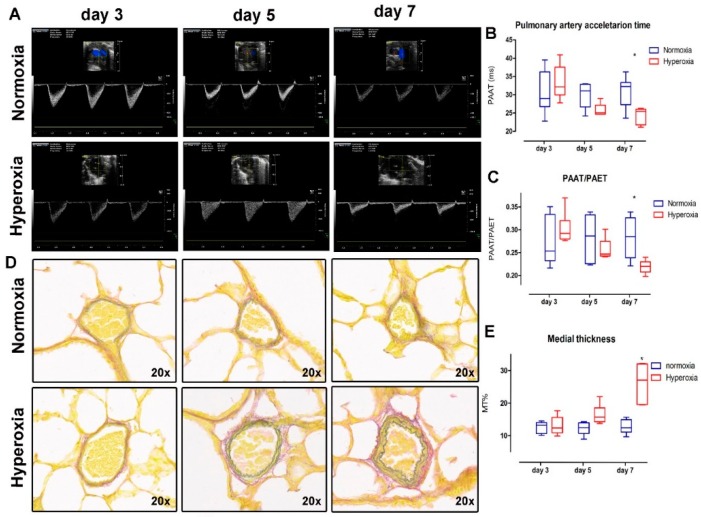
Pulmonary artery micro-ultrasound and lung peripheral arteries media thickness: (**A**) Representative images of micro-ultrasound scans of pulmonary artery Doppler showing a progressive decrease of pulmonary artery acceleration time (PAAT) and PAAT/pulmonary artery ejection time (PAET) ratio in the hyperoxia group; (**B**) PAAT; (**C**) PAAT/PAET; (**D**) Representative images of lung peripheral arteries showing progressive thickening of tunica media in hyperoxia group pups; (**E**) Media thickness (MT%) of lung peripheral arteries. * = Significant interaction between time and group in two-way ANOVA; significant difference between studied groups in day seven. *n* = 6 per time point in each group.

**Figure 2:**
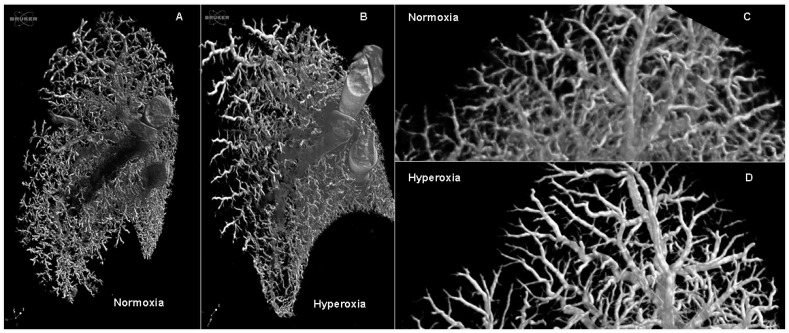
Representative images of Micro-CT of barium angiograms: Barium angiograms showed less distal vascular branches on pups exposed to hyperoxia: (**A**) Barium angiogram of a left lung of a normoxia exposed pup on day seven; (**B**) Barium angiogram of a left lung a hyperoxia-exposed pup on day seven; (**C**) higher detail of Panel A; (**D**) higher detail of Panel B.

Functional vascular assessment of the main pulmonary artery showed a decreased pulmonary artery acceleration time (PAAT) (Figure 1B) (interaction *p* = 0.017; hyperoxia *p* = 0.01; time *p* = 0.04; subjects matching *p* = 0.74). In the multiple comparison measurements on day three and five, the data were not different (day three mean difference: 2.43, 95% confidence interval (CI): −3.1 to 7.97; day five mean difference: −4.98, CI: −10.53 to 0.55), yet measurements were significantly different on day seven (day seven mean difference: −7.49, CI: −13.04 to −1.95). The PAAT/pulmonary artery ejection time (PAET) ratio (Figure 1C) decreased over time, but it did not show an effect of hyperoxia exposure between groups (interaction *p* = 0.007; hyperoxia *p* = 0.08; time *p* = 0.03; subjects matching *p* = 0.12). The multiple comparison measurements on day three and five were not different (day three mean difference: 0.02, 95% confidence interval (CI): −0.03 to 0.07; day five mean difference: −0.03, CI: −0.08 to 0.01), but they were significantly different on day seven (day seven mean difference: −0.07, CI: −0.12 to −0.01). At the histological level, the peripheral arterial media thickness was significantly increased in the hyperoxia cohort (MT%; Figure 1E) (interaction *p* < 0.0001; hyperoxia *p* < 0.0001; time *p* < 0.001). In the multiple comparison of the data on day, three and five were not different (day three mean difference: 0.07, 95% confidence interval (CI): −4.57 to 4.71; day five mean difference: 4.47, CI: −0.16 to 9.11), yet they were significantly different on day seven (day seven mean difference: 13.07, CI: 8.42 to 17.71). Barium angiograms showed less ‘distal vessel density’ in hyperoxia-exposed pups (Figure 2).

Invasive pressure measurements on seven-day-old term pups confirmed the inverse correlation of the PAAT and PAAT/PAET ratio and a higher systolic peak pressure in the right ventricle, as seen in the mouse model [16] and in humans with pulmonary hypertension [17] (Figure S1).

### 2.2. Functional Parenchymal Changes: Hyperoxia Causes Progressive Functional Changes in the Lung Parenchyma of Preterm Rabbits

**Figure 3:**
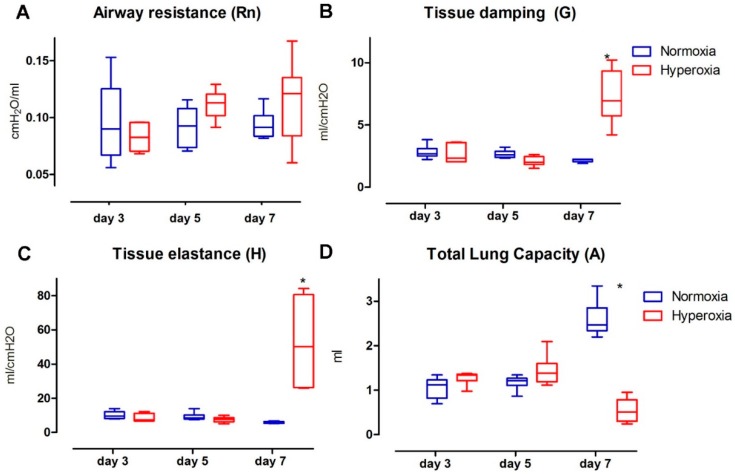
Flexivent results: (**A**) Airway resistance (Rn); (**B**) Tissue damping (G); (**C**) Tissue elastance (H); (**D**) Total lung capacity (A). * = Significant interaction between time and group in two-way ANOVA; significant difference between studied groups in day seven. *n* = 6 per time point in each group.

Hyperoxia induced significant and progressive changes in invasive parenchymal functional parameters, while airway resistance (Rn) was not affected by hyperoxia exposure over the studied period (Figure 3A). Rn values did not change over time and were not affected by hyperoxia (interaction *p* = 0.152; hyperoxia *p* = 0.262; time *p* = 0.275).

Tissue damping (G; Figure 3B) showed a significant increase between the study groups (interaction *p* < 0.0001; hyperoxia *p* < 0.0001; time *p* < 0.0001). In the multiple comparison analysis, the data on day three and five were not different (day five mean difference: −0.144, 95% confidence interval (CI): −1.56 to 1.27; day 5 mean difference: 0.575, CI: −1.99 to 0.84), but they were significantly different on day seven (mean difference: 5.1, CI: 3.68 to 6.52). Tissue elastance (H; Figure 3C) was significantly higher in the hyperoxia group (interaction *p* < 0.0001; hyperoxia *p* = 0.002; time *p* = 0.0004). In the multiple comparison, the values of day three and five were not different (day three mean difference: −1.583, 95% CI: −14.25 to 17.41; day five mean difference: −1.676, CI: −14.16 to 17.51), yet they were significantly different on day seven (mean difference: 46.79, CI: 30.96 to 62.63).

Total lung capacity (A; Figure 3D) was significantly lower in the hyperoxia group (interaction *p* < 0.0001; hyperoxia *p* < 0.0001; time *p* = 0.003). In the multiple comparison analysis, data on day three and five were not different (day three mean difference: −0.14, 95% confidence interval (CI): −1.56 to 1.27; day five mean difference: 0.57, CI: −1.99 to 0.84), but significantly different on day seven (day seven mean difference: 5.1, CI: 3.68 to 6.52). Static compliance (Cst; Table 1) was also decreased in the hyperoxia cohort (interaction *p* < 0.0001; hyperoxia *p* < 0.0001; time *p* = 0.004). In the multiple comparison analysis, day three and five were not different (day three mean difference: 0.22, 95% confidence interval (CI): −0.17 to 0.62; day five mean difference: 0.33, CI: −0.06 to 0.73), but again a significant difference was present on day seven (day seven mean difference: −2.04, CI: −2.44 to −1.64).

### 2.3. Structural Changes in the Lung Parenchyma: Vascular Changes Are Related to Progressive Structural Changes in the Lung Parenchyma of Preterm Rabbits

**Figure 4:**
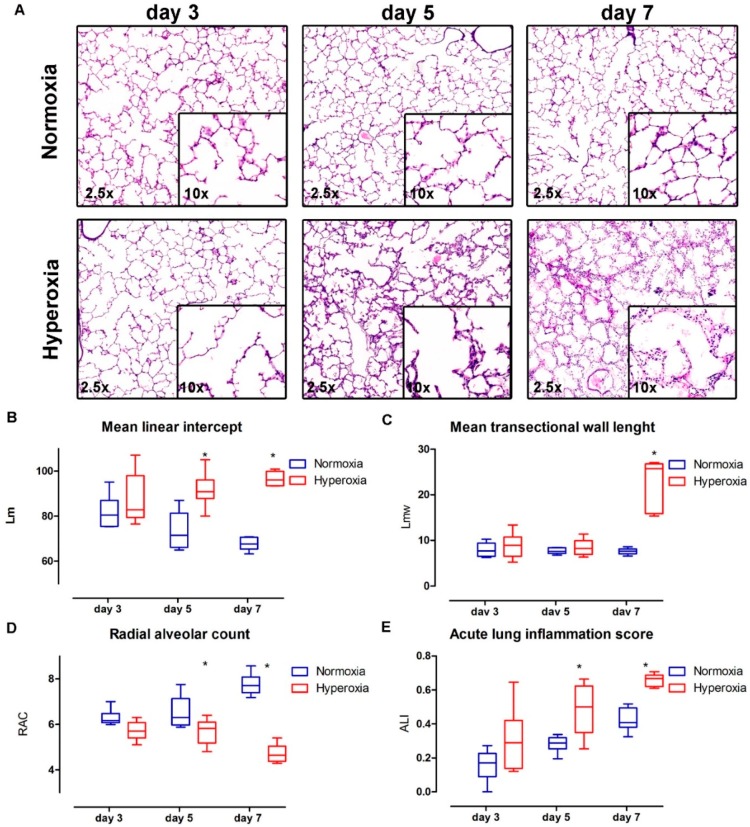
Lung parenchyma histology results: (**A**) Representative images of H&E lung sections at 2.5× and 10× magnification. Hyperoxia-exposed pups showed larger and fewer alveoli, with thicker alveolar walls, less airway complexity and a higher inflammation score; (**B**) Mean linear intercept (Lm; alveolar size); (**C**) Mean transectional wall length (Lmw; interalveolar septum thickness); (**D**) Radial alveolar count (RAC); (**E**) Acute lung inflammation score (ALI). * = Significant interaction between time and group in two-way ANOVA; significant difference between studied groups in day five and seven. *n* = 6 per time point in each group.

The lung histology (Figure 4A) results showed an increased alveolar size in the pups exposed to hyperoxia (Lm; Figure 4B) (interaction *p* = 0.002; hyperoxia *p* < 0.0001; time *p* = 0.68). In the multiple comparison, day three was not different (day three mean difference: 5.52, 95% confidence interval (CI): −5.46 to 16.52), yet this became significant on day five and seven (day five mean difference: 18.24 CI: 7.24 to 29.23; day seven mean difference: 28.92, CI: 17.93 to 39.92). Hyperoxia-exposed pups had thicker alveolar walls (Lmw; Figure 4C) (interaction *p* < 0.0001; hyperoxia *p* < 0.0001; time *p* < 0.0001). In the multiple comparison, day three and five data were not different (day three mean difference: 0.93, 95% confidence interval (CI): −3.07 to 4.93; day five mean difference: 0.76, CI: −3.23 to 4.77), yet day seven data were significantly different (day seven mean difference: 15.15, CI: 11.15 to 19.16). The radial alveolar count (RAC; Figure 4D), reflecting lung parenchyma maturation, was consistent with these results, and showed a decreased airway complexity in hyperoxia-exposed pups (interaction *p* < 0.001; hyperoxia *p* < 0.0001; time *p* = 0.53). In the multiple comparison, day three was not different (day three mean difference: 0.57, 95% confidence interval (CI): −0.16 to 1.3), yet a significant difference was detected on day five and seven (day five mean difference: 0.83 CI: 0.09 to 1.57; day seven mean difference: 3.04, CI: 2.3 to 3.77). Lung parenchymal inflammation increased over time in both normoxia and hyperoxia, but it was significantly higher the hyperoxia group (acute lung inflammation score; Figure 4E) (interaction *p* = 0.64; hyperoxia *p* < 0.0001; time *p* < 0.0001). In the multiple comparison, day three was not different (day three mean difference: 0.14, 95% confidence interval (CI): −0.01 to 0.31), but significant differences were detected on day five and seven (day five mean difference: 0.2 CI: 0.03 to 0.37; day seven mean difference: 0.23 CI: 0.06 to 0.4).

## 3. Discussion

“New” BPD is currently viewed as a disruption of lung development with decreased septation and alveolar hypoplasia, but also a dysregulated lung vasculature development that results in increased pulmonary resistance. In order to study this disease, animal models are imperative as a histological, molecular or radiological micro-architectural analysis is not always feasible in humans. The rabbit is a model with a lung development that is much closer to humans compared to mice and rats, which on its own is a favorable characteristic. Additionally, rabbits are not as expensive and ethically challenging as large animal models, so they are ideally suited for the testing of experimental strategies, be it pharmacological agents or even gene- and stem cell therapies. Yet, in this context, the model itself requires a more thorough analysis of all its aspects. We previously described the parenchymal changes [15] and a transcriptome analysis [18], yet a detailed assessment of the vascular changes was still lacking. In this study we documented very comprehensively the progressive functional and histological hyperoxia-induced damage in the lung vasculature.

We determined that it takes seven days of hyperoxia-exposure to induce a significant difference compared with normoxia in the studied parameters. At this time point, we found a significant functional vascular damage, with shortening of both PAAT and the PAAT/PAET ratio. This was reflected at histological level with an increased thickness of the media layer of intra-acinar pulmonary arteries. Our findings that PAAT and PAAT/PAET ratio (parameters that are inversely related to the right ventricle systolic pressure (RVSP) [16]) decreased as the hyperoxia exposure time was extended demonstrates that the non-invasive assessment of pulmonary artery systolic pressure is feasible and reproducible. It was not possible to measure the actual pressure in hyperoxia-exposed pups, due to the intolerance of the animals to prolonged sedation or anesthesia. Instead we used term rabbit pups—where invasive pressure measurements are feasible—and showed the correlation between the shorter PAAT and the PAAT/PAET ratio and a higher RVSP. The latter was induced by U-46619 infusion, a pulmonary vasculature constrictor. Vascular hyperoxia damage in this model was associated with similar changes to what is described in human BPD. Early echocardiographic signs (seven days after preterm birth) of pulmonary hypertension were present in 42% of 277 preterm infants who had PH and BPD as measured at 36 corrected gestational age (GA) weeks in 21% to 84% of cases. In the absence of early PH signs, BPD was only present in 10% of that group, showing that early pulmonary vascular disease in preterm infants contributes to BPD morbidity [19]. In other animal models, the presence of vascular changes is variable. In baboons, known to have a similar perinatal lung development and lung anatomical features as humans, no vascular changes have been described [13,20]. At present, the use of the baboon model is very difficult due to ethical concerns and the high expenses. In newborn rodents, hyperoxia leads to alveolar simplification and decreased vessel density, with functional lung and vascular changes that are similar to that of human [12]. In the same species, angiogenesis blockade by vascular endothelial growth factor (VEGF)-Trap decreased the number of lung capillaries and increased the size of alveoli, similar to BPD. This suggests that angiogenesis participates in alveolarization [21]. Treatment with bone marrow-derived mesenchymal stem cells of newborn rats exposed to hyperoxia (95% for 14 days) corrected both the alveolar arrest and the vascular damage, measured with PAAT, vessel density and proliferation [22]. This is in line with the concept that the parenchyma and the vascular compartment are closely interconnected and that assessment of both is necessary to understand the potential benefit of novel preventive and therapeutic interventions.

In this study, we also detailed the progression of lung parenchymal damage in the model, by describing functional and histological changes at day three, five and seven. At the functional level, tissue resistance, elastance, compliance and total lung capacity were affected after hyperoxia exposure. But there were no airway resistance changes during this period, as is seen in early human BPD [23]. In preterm infants, early lung function at day three after birth is more severely affected in those who will develop moderate/severe BDP. Those infants had lower lung volumes (measured by functional residual capacity) and compliance, yet no higher airway resistance than infants who did not develop BPD [24]. This suggests that the functional changes induced by hyperoxia in preterm rabbits are similar to the early changes in BPD affected infants, adding valuable information in this model for the study of preventive and early therapeutic interventions for BPD. Lung parenchymal histological changes in hyperoxia showed significant changes in alveolar size and number (lower Lm and RAC) with thicker alveolar wall in this model. In BPD, affected infants have fewer and larger alveoli [3,25]. This is a prerequisite in an animal model that would be considered as a BPD model [10,11,12,13,14,26]. It has been described that preterm hyperoxia-exposed rabbit pups have larger alveolar size and fewer alveoli after 7 and 11 days of hyperoxia (95%). In addition they also displayed an impaired lung function, including decreased total lung capacity, static compliance and increased tissue damping and elasticity [15,27]. The reported survival after 7 and 11 days was 56% and 11%, respectively. Those data are in line with our results.

As hyperoxia is the main stimulus that causes the pathophysiological changes in this model, it is important to review the available knowledge in the rabbit. It has been shown that preterm rabbits are less resistant to hyperoxia exposure [28] than term pups [29], especially with regard to survival and lung antioxidant enzyme activity. Surfactant and antioxidant systems develop quickly during the last three to five days of gestation in rabbits (term 31 days), as they mature from early saccular to the alveolar phase [30,31]. The preterm rabbit fails to upregulate catalase and superoxide dismutase (enzymes related to oxidative damage) when exposed to hyperoxia, as term pups do [32]. It is believed that the free oxygen radicals cause tissue damage, which in turn leads to an inflammatory response. We assessed inflammation using a histological score proposed by the American Thorax Society [33]. The inflammation increases in normoxia between days three and seven, but is even higher in the hyperoxia group. There are only very limited data on the presence of immune cells in fetal and postnatal lungs. It seems that the presence of immune cells increases in airways and lung parenchyma of both human and monkey infants even in non-pathological conditions [34,35], yet they are always higher in case of infection or inflammation.

In conclusion, our current study provides new insights in the preterm-hyperoxia rabbit model as it consists of very comprehensive functional and histological vascular analyses that can be used to validate other findings in the same model. We recently reported a transcriptome analysis by mRNa sequencing in this model, and detected 2217 dysregulated transcripts following hyperoxia [18]. In that study several relevant pathways were identified, involving lung development, acute inflammation but also lung vasculogenesis. Based on those data, we hypothesized the presence of the functional and structural vascular changes reported here, which strengthens the translational value of this model.

## 4. Materials and Methods

### 4.1. Animal Protocols

Time-mated pregnant rabbits (New Zealand White and Dendermonde hybrid) were provided by the animalium of the group Biomedical Sciences at the KU Leuven. The experiments were approved by the Ethics committee for Animal Experimentation of the Faculty of Medicine (p107/2013) and performed according to current guidelines on animal welfare. Does were housed in separate cages before cesarean section at 28 days of gestational age (GA) of pregnancy (term = 31 days GA).

Ten does were sedated with intramuscular ketamine 35 mg/kg bodyweight (BW) (Ketamine 1000 CEVA; CEVA Santé Animal, Brussels, Belgium) and xylazin 6 mg/kg BW (Vexylan^®^; CEVA Santé Animal, Brussels, Belgium). Subsequently, spinal anesthesia with 2% lidocain hydrochloride (Linisol^®^; B Braun, Brussels, Belgium) at the level of L7–S1 was given for cesarean section. The does were placed in the supine position, and the abdomen was opened through a low midline abdominal incision. The uterus was exposed, and all pups were extracted through hysterotomy. Following delivery, the does were euthanized with a mixture of 200 mg embutramide, 50 mg mebezonium, and 5 mg tetracain hydrochloride (iv. bolus of 1 mL T61^®^; Intervet; Belgium, Mechelen, Belgium).

At delivery, the pups were dried, stimulated and placed in an incubator (Dräger Incubator 7310; Dräger, Lübeck, Germany) at 32 °C and 75% of humidity. Oxygen concentration was continuously monitored with a Palm O_2_ D% Analyser^®^ (Analytical Industries; Pomona, Claremont, CA, USA). After one hour, the survivors were weighed and numbered. Thereafter pups were fed twice per day via a 3.5 Fr orogastric tube placed just before the feeding. The feeding consisted of a milk replacer (Day One^®^, Protein 30%, Fat 50%; FoxValley; IL, USA) mixed with water according to the manufacturer’s directions. Probiotics, electrolytes and vitamins were added during the first 5 postnatal (PN) days (Bio-Lapis^®^; Probiotics International Ltd.; Somerser, UK) and immunoglobulins during the first 2 PN days (Col-o-Cat^®^, SanoBest; Hertogenbosch, The Netherlands). The amount of feeding steadily increased from 80 to 100 to 150 to finally 200 mg/kg BW day on PN days 0, 1, 2, and 3 (until 7), respectively. On PN day 2, vitamin K1 was administered intramuscularly (0.002 mg/kg BW, Konakion pediatrique^®^; Roche, Basel, Switzerland), and from that point until harvest, pups were given a daily intramuscular injection of benzylpenicillin (20,000 I.U./kg BW Penicilline^®^; Kela, Sint-Niklaas, Belgium) and amikacin (20 mg/kg BW day, Amukin^®^; Bristol-Myers-Squibb, Brussels, Belgium). The pups remained in the incubator except for the feeding and ultrasound examination, for which they were removed from the incubator for a maximum of 5 min. The nests were randomly assigned to: (1) normoxia group (21% oxygen); and (2) hyperoxia group (≥95% oxygen). Stratified random sampling on surviving pups at PN day 3, 5 and 7 was used to create blocks of pups to be harvested. A total number of 36 pups were used for this study, 6 per groups in each studied time point. They were tested will all the studied parameters described below. All assessments were done by two independent observers blinded to the cohort allocation. Three additional pups per group were used for barium angiograms.

### 4.2. Pulmonary Artery Micro Ultrasound Doppler

Pups were removed from the incubator and take to the examination area within the same room and were sedated with 3% isoflurane in a mixture of 100% O_2_ at 2 L/min until spontaneous movement decreased and then lowered to 2% during the measurements, within an average time of 3 min per pup. Transthoracic closed chest echocardiography was performed daily from PN day 3, 5 and 7 in 6 pups per group by Julio Jiménez, as described before [16], briefly two-dimensional images of the pulmonary infundibulum were obtained from a parasternal short axis view at the level of the aortic valve using a mechanical transducer (30 MHz, Vevo 2100; Visualsonics, Toronto, ON, Canada). The pulse-wave Doppler sample was positioned at the tip of the pulmonary valve leaflets and aligned. Sample volume was 0.027 mm^3^ and recorder at a sweep speed of 400 mm/s. Measurements were performed offline (Vevo 2100 software package V1.5.0, Toronto, ON, Canada). The following variables were measured: pulmonary artery acceleration time (PAAT), ejection time (PAET) and PAAT/PAET ratio. An average on five cardiac cycles was use for the analyses. Pups who died before reaching PN day 7 were excluded from the analysis (i.e., 2 in hyperoxia group).

### 4.3. Invasive Measurements

PAAT, PAET and PAAT/PAET ratio is a validated method to estimate pulmonary artery pressure in mice and humans [17,36]. We performed invasive right ventricular systolic pressure (RVSP) measurement in two healthy rabbit pups to confirm this correlation in newborn rabbits. Term pups were caged in normoxia and fed during 7 days after cesarean section as described above in the text. Rabbits were placed on a warm plate and sedated with 3% isoflurane in a mixture of O_2_ at 2 L/min. Local anesthesia with 2% lidocain hydrochloride (Linisol^®^; B Braun) was injected in the anterior aspect of the neck. A 2.5 Fr catheter (Argyle^®^ 2.5 Fr, Covidien, Ireland) was inserted into the right jugular vein and advanced into the right ventricle. The tip’s position in the right ventricle was confirmed by ultrasound view. A second 2.5 Fr catheter was inserted in the right jugular vein for infusion of the vasoconstrictor U-46619 (U-46619, Tocris; Bristol, UK). RVSP was recorded continuously. Simultaneously, PAAT and PAET was measured as described above. U-46619 was then infused at a rate of 1.5 µmol/kg/min for 10 min and both measurements were obtained. The infusion was discontinued for 15 min, and after the RVSP returned baseline, U-46619 was infused at 3 µmol/kg/min rate for a second set on measurements [16].

### 4.4. Lung Function Test

The invasive lung function test was done using the Flexivent system (FlexiVent 5.2; SCIREQ; Montreal, QC, Canada) as previously described [15]. Six pups were measured from each group on PN day 3, 5 and 7. After anesthesia with ketamine 35 mg/kg BW (Ketamine 1000 CEVA; CEVA Santé Animal, Brussels, Belgium) and xylazin 6 mg/kg BW (Vexylan^®^; CEVA Santé Animal), an 18 G metal needle was inserted in the trachea and connected to the Flexivent (120 breaths/min ventilation; Module 1 or 2 if pup weight < or >40 g respectively; PEEP 3 cm H_2_O). Primewave-8 perturbation (P8) was used to assess airway resistance (Rn), tissue damping (G; frequency independent parenchymal energy dissipation), tissue elastance (H; energy conservation of lung tissue). Pressure volume perturbation (PVr-V) was used to assess total lung capacity (A; estimation of inspiratory capacity) and static compliance (Cst; elastic recoil pressure of the lung at a given lung volume). Three measures were obtained per pup (coefficient of determination >0.95) and the mean value was used in the analyses. After this, pups were euthanized with 0.1 mL of intravascular T61^®^.

### 4.5. Histological Assessment of the Lungs

After euthanasia, a thoracotomy was performed and the lungs were removed “in bloc”. A 20-G catheter was inserted in the trachea and the left lung was fixed with 4% paraformaldehyde by immersion and under a constant hydrostatic pressure of 25 cm H_2_O for 24 h before embedding. Paraffin sections were stained with hematoxylin and eosin (H&E) and Miller’s elastic stain. Lung morphometric measurements were done in 20 random fields as described before, including mean linear intercept (alveolar size; L_m_), and mean wall transection length (inter-alveolar septal thickness; L_mw_) [37]. Radial alveolar count (RAC) was measured at least 20 times per animal lung as described before [37,38]. For vascular morphometry, at least 15 arteries under 100 µm of external diameter were measured to obtain the internal (ID) and external (ED) diameter of the media (muscular) layer [39]. A lung injury scoring system (LIS) proposed by the American Thorax Society [33] was used to compare lung tissue inflammation in both groups using 20 random high power fields (400×) over lung alveoli in five histological findings (i.e., neutrophils in the alveolar space, neutrophils in the interstitial space, hyaline membranes, proteinaceous debris in the airspace and alveolar septal thickening).

### 4.6. Barium Angiograms

Three pups per group were randomly selected on PN 7 for lung barium angiograms. A barium-gelatin-thymol (BGT) mixture with 8.4% gelatin and 3% of thymol (Merk; Darmsadt, Germany) in barium sulfate (Micropaque; Guerbet, Suizbach, Germany) at 37 °C was infused via the right jugular vein, as previously described [40]. Animals were cooled in at 4 °C and the lungs were harvested in bloc when BGT was solid. Samples were fixed in 4% paraformaldehyde and kept at 4 °C overnight. The lungs were sectioned in left and right, and were scanned in a nano-CT unit (Phoenix nanoTom, General Electric; Wunstorf, Germany) using 60 keV, 190 µA, mode 0 no filter, exposure time 500 ms, 600 images, voxel size 10 µm. Images were rendered using CTVox (Brucker; Aartselaar, Belgium).

### 4.7. Statistical Analysis

Sample size was calculated with G*Power 3.1.9.2 software (Kiel University, Kiel, Germany) with an effect size of 0.5, α error = 0.01, 2 groups, and 3 measurements based on previous results in this model [15]. Statistical analyses were done with GraphPad Prism 7.0 software (GraphPad; La Jolla, CA, USA). Continuous variables were analyzed with 2-way ANOVA, using time and group allocation as factors. Bonferroni posttest was used to compare groups for each day, reporting results as difference of means of studied groups and 95% confidence intervals. Linear regression was used to correlate RVSP with ultrasound measurements. A value of *p* < 0.05 was considered statistically significant. All values are expressed as mean and SD.

## 5. Conclusions

Hyperoxia induces progressive functional and structural vascular damage in preterm rabbits, mimicking the early onset of bronchopulmonary dysplasia in humans. This model enables the testing of pharmaceutical agents that target the cardiovascular compartment of the lung for further translation towards the clinic.

## Figures and Tables

**Table 1:** Two-way analysis of variance (ANOVA) results.

Parameter	Measurment	Normoxia			Hyperoxia		
		Day 3	Day 5	Day 7	Day 3	Day 5	Day 7
Pulmonary Artery Ultrasound and Histology	Pulmonary artery acceleration time (PAAT) ms ± SD ^1^	31.73 ± 6.682	31.28 ± 2.687	32.34 ± 3.240 ^2^	33.5 ± 5.17	25.69 ± 1.859	25.03 ± 1.749 ^2^
	Pulmonary artery acceleration/ejection time ratio (PAAT/PAET)% ± SD ^3^	28.47 ± 5.34	30.48 ± 3.128	29.92 ± 3.653 ^2^	30.58 ± 3.742	26.03 ± 2.478	24.19 ± 3.229 ^2^
	Media thickness % (MT%) ^4^	12.57 ± 1.827	12.42 ± 2.077	12.83 ± 2.171 ^2^	13.12 ± 5.059	14.56 ± 3.325	16.58 ± 2.979 ^2^
Flexivent Parameter	Airway resistance (Rn) cmH_2_O/mL ± SD	0.095 ± 0.014	0.091 ± 0.007	0.093 ± 0.005	0.082 ± 0.004	0.111 ± 0.005	0.124 ± 0.016
	Tissue damping (G) cmH_2_O/mL ± SD ^4^	2.736 ± 0.474	2.630 ± 0.351	2.162 ± 0.133 ^2^	2.665 ± 0.742	2.079 ± 0.387	7.069 ± 1.967 ^2^
	Tissue elastance (H) cmH_2_O/mL ± SD ^4^	10.40 ± 3.116	9.314 ± 2.655	5.927 ± 0.533 ^2^	8.551 ± 2.379	7.512 ± 1.671	49.25 ± 8.5 ^2^
	Total lung capacity (A) mL ± SD ^5^	1.1 ± 0.186	1.178 ± 0.163	2.593 ± 0.401 ^2^	1.278 ± 0.149	1.455 ± 0.324	0.545 ± 0.256 ^2^
	Static compliance (Cst) mL/cmH_2_O ± SD ^6^	0.051 ± 0.013	0.061 ± 0.008	0.134 ± 0.021 ^2^	0.067 ± 0.015	0.078 ± 0.033	0.025 ± 0.01 ^2^
Histology Parenchymal	Linear intercept (Lm) ± SD ^7^	81.87 ± 7.342	73.48 ± 9.204	67.66 ± 2.883	87.39 ± 11.45	91.71 ± 8.006	96.58 ± 5.229
	Mean wall thickness (Lmw) ± SD ^4^	7.927 ± 1.56	7.673 ± 0.676	7.609 ± 0.691 ^2^	8.859 ± 2.788	8.441 ± 1.818	22.76 ± 5.35 ^2^
	Radial alveolar count (RAC) ± SD ^8^	6.287 ± 0.365	6.528 ± 0.711 ^2^	7.756 ± 0.471 ^2^	5.717 ± 0.415 ^2^	5.692 ± 0.562 ^2^	4.715 ± 0.418 ^2^
	Acute lung inflammation score (ALI) ± SD ^9^	0.157 ± 0.093	0.281 ± 0.049 ^2^	0.423 ± 0.069 ^2^	0.305 ± 0.188	0.484 ± 0.159 ^2^	0.659 ± 0.036 ^2^

^1^ Repeated measures two-way ANOVA: Interaction *p* = 0.017; hyperoxia *p* = 0.01; time *p* = 0.04; subjects matching *p* = 0.74; ^2^ *p* < 0.001; ^3^ Repeated measures two-way ANOVA: Interaction *p* = 0.007; hyperoxia *p* = 0.08; time *p* = 0.03; subjects matching *p* = 0.12; ^4^ Interaction, time and group allocation *p* < 0.001; ^5^ Interaction and group allocation *p* < 0.0001, time *p* = 0.002; ^6^ Interaction and group allocation *p* < 0.0001, time = 0.028; ^7^ Interaction and group allocation *p* < 0.0001, time *p* = 0.5; ^8^ Interaction *p* = 0.3, group allocation and time *p* < 0.0001; ^9^ Repeated measures two-way ANOVA: Interaction *p* = 0.007; hyperoxia *p* = 0.08; time *p* = 0.03; subjects matching *p* = 0.12.

## Supplementary Materials

Supplementary materials can be found at www.mdpi.com/1422-0067/17/10/1776/s1.

## Abbreviations

A	Total lung capacity
BPD	Bronchopulmonary Dysplasia
BW	Body weight
Cst	Static compliance
ED	External diameter
G	Tissue damping
GA	Gestational age
H	Tissue elastance
H&E	Hematoxylin and eosin
ID	Internal diameter
LIS	Lung injury score system
Lm	Mean linear intercept
Lmw	Mean wall transection length
P8	Primewave-8 perturbation
PAAT	Pulmonary artery acceleration time
PAET	Pulmonary artery ejection time
PAAT/PAET ratio	Pulmonary artery acceleration/ejection time ration
PN	Postnatal
PVr-V	Pressure volume perturbation
RAC	Radial alveolar count
Rn	Airway resistance
RVSP	Right ventricular systolic pressure
