# Supplementary material for: Progressive Vascular Functional and Structural Damage in a Bronchopulmonary Dysplasia Model in Preterm Rabbits Exposed to Hyperoxia
Source: Int J Mol Sci. 2016 Oct 24;17(10):1776. doi: 10.3390/ijms17101776 (PMC5085800; doi:10.3390/ijms17101776)
Supplement: Supplementary file 1 [file ijms-17-01776-s001.pdf]

Supplementary Materials: Progressive Vascular Functional and Structural Damage in a Bronchopulmonary Dysplasia Model in Preterm Rabbits Exposed to Hyperoxia

Julio Jimenez, Jute Richter, Taro Nagatomo, Thomas Salaets, Rozenn Quarck, Allard Wagennar, Hongmei Wang, Jeroen Vanoirbeek, Jan Deprest and Jaan Toelen

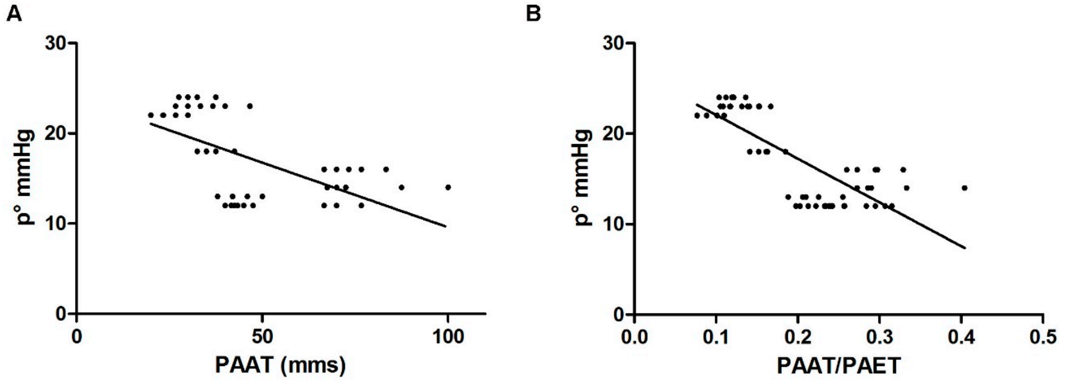

Figure S1. Correlation between the invasive measurement of the right ventricle systolic peak pressure and the pulmonary artery Doppler micro-ultrasound measurements (**A**) Linear regression of the right ventricle systolic peak pressure and the pulmonary artery acceleration time measured by Doppler. y = −0.1433x + 23.926; R^2 = 0.329; (**B**) Linear regression of the right ventricle systolic peak pressure and the pulmonary artery acceleration time and ejection time ratio measured by Doppler. y = −48.35x + 26.91; R^2 = 0.621. *n* = 2.
